# The Frontal QRS-T Angle Remains Unchanged in Patients Without Structural Heart Disease Receiving Flecainide Therapy

**DOI:** 10.3390/jcdd13040167

**Published:** 2026-04-14

**Authors:** Mehmet Kucukosmanoglu, Mustafa Lutfullah Ardıc, Fadime Koca, Hilmi Erdem Sumbul, Mevlut Koc

**Affiliations:** 1 Department of Cardiology, University of Health Sciences, Adana Health Practice and Research Center, Adana 01230, Turkey; drmustafaardic@gmail.com (M.L.A.); mevlutkoc78@yahoo.com (M.K.); 2 Department of Cardiology, Cukurova State Hospital, Adana 01170, Turkey; drfadimekoca@gmail.com; 3 Department of Internal Medicine, University of Health Sciences, Adana Health Practice and Research Center, Adana 01230, Turkey; erdemsumbul@gmail.com

**Keywords:** flecainide, QRS-T angle, arrhythmia

## Abstract

**Introduction:** Prolongation of the QT interval and QRS duration, which are markers of ventricular repolarization and depolarization, has been reported in patients receiving flecainide therapy. However, the effects of flecainide on the QRS–T angle—a recognized indicator of transmural dispersion of repolarization—remain unclear. The aim of our study was to investigate the impact of flecainide therapy on the QRS–T angle. **Method:** In this study, 200 patients who were prescribed flecainide therapy due to atrial or ventricular arrhythmias were included. Prior to the initiation of flecainide treatment, all patients underwent a 12-lead electrocardiogram (ECG) in which heart rate (HR), PR and QRS durations, QT, QTc, JT, Tp–Te intervals and the frontal plane QRS–T angle were measured. At the 1-month follow-up, patients underwent repeat ECG recording and were evaluated for both cardiac and non-cardiac side effects of flecainide. The same ECG parameters were measured again using the follow-up recordings. Changes in ECG parameters between the baseline and 1-month post-treatment were analyzed. **Results:** Following flecainide administration, the drug was discontinued in 18 patients (9%) due to adverse effects (11 cases of cardiac and seven cases of non-cardiac). HR significantly decreased (78 ± 22 bpm to 74 ± 15 bpm and *p* < 0.05). PR interval and QRS duration significantly increased (148 ± 23 ms to 156 ± 9 ms and 89 ± 17 ms to 99 ± 19 ms, respectively *p* < 0.05 for each). Additionally, JT interval (326 ± 27 ms vs. 334 ± 6 ms), QT interval (416 ± 24 ms vs. 434 ± 24 ms), QTc interval (431 ± 24 vs. 447 ± 25 ms) and Tp–Te interval (84 ± 17 vs. 87 ± 18 ms) all showed statistically significant increases after flecainide treatment (*p* < 0.05 for-each). However, no significant change was observed in the frontal QRS–T angle. **Discussion:** In patients receiving flecainide therapy for atrial and ventricular arrhythmias, prolongation was observed in atrioventricular conduction, ventricular depolarization and repolarization parameters as measured by ECG. However, no significant change was detected in the frontal QRS–T angle.

## 1. Introduction

Flecainide acetate is a class IC antiarrhythmic drug (AAD) and a potent inhibitor of the Nav1.5 sodium channel. It was approved for clinical use by the U.S. Food and Drug Administration in 1985. In patients without structural heart disease, flecainide is recommended for the treatment of supraventricular arrhythmias—including atrial fibrillation, atrioventricular nodal reentrant tachycardia, atrioventricular reentrant tachycardia and atrial premature beats—as well as certain ventricular arrhythmias such as ventricular tachycardia, premature ventricular contractions and selected channelopathies [1,2,3,4].

Numerous electrocardiographic (ECG) parameters are associated with ventricular depolarization and repolarization and many of these parameters have been linked to malignant ventricular arrhythmias and prognosis in both cardiac and non-cardiac conditions [5,6,7]. The QRS–T angle reflects the spatial dispersion of ventricular repolarization and has been shown to increase in the presence of various cardiovascular diseases and risk factors, with elevated values being associated with adverse cardiovascular events [8,9,10,11,12,13,14,15,16,17,18].

As with other class IC agents, flecainide reduces the amplitude of the action potential, prolongs phase 0 depolarization, and extends the overall action potential duration [19]. These changes also result in prolongation of the repolarization phase [19]. Several studies have reported that flecainide therapy leads to the prolongation of repolarization parameters such as JT [20], QT [1,19,20,21,22], QTc [1,19,20,21,22] and Tp–Te intervals [21,22], as well as depolarization markers such as the QRS duration [1,19,20,21,22,23,24].

In routine clinical practice, patients initiated on flecainide therapy undergo standard electrocardiographic (ECG) evaluation during the second week of treatment to assess for drug-related adverse effects. Among these, prolongation of the QRS duration—a marker of depolarization—represents the most clinically significant adverse effect [1,23,24] and constitutes the leading cause of treatment discontinuation [3,4]. However, flecainide may induce the prolongation of both depolarization and repolarization parameters. The frontal QRS-T angle, calculated as the absolute difference between the QRS axis and the T axis, is an electrocardiographic parameter that integrates both domains. Given flecainide’s established effect on the QRS duration, it was hypothesized that the drug may also exert a measurable influence on the frontal QRS-T angle. However, the effect of flecainide on the frontal QRS–T angle, which is considered a marker of transmural dispersion of repolarization, remains unknown.

The aim of our study was to investigate the effect of flecainide therapy on the frontal QRS–T angle in patients with atrial and ventricular arrhythmias.

## 2. Methods

### 2.1. Study Population

This study was designed as a single-center, retrospective, cross-sectional analysis. Patients who presented to the Arrhythmia Outpatient Clinic of Adana City Training and Research Hospital between August 2022 and May 2025 with supraventricular or ventricular arrhythmias and were considered for antiarrhythmic therapy were screened. A total of 245 patients who received flecainide as antiarrhythmic treatment were identified. To determine the required sample size, a power analysis was conducted based on prior studies and expected outcomes (80% power, *p* < 0.05). The analysis indicated that including approximately 100 patients would be sufficient. After applying exclusion criteria, 200 out of the 245 patients were included in the final analysis.

Exclusion criteria were as follows: age ≤ 18 years, coronary artery disease, hypertensive heart disease, second- or third-degree atrioventricular block, isolated left or right bundle branch block, ongoing atrial fibrillation or flutter, prior ablation therapy for atrial or ventricular arrhythmias, use of antiarrhythmic medication within the past 5 days, severe valvular heart disease, congenital heart disease, significant renal or hepatic impairment, electrolyte imbalances, thyroid dysfunction, pregnancy or postpartum period within 3 months, chronic inflammatory disease, or active malignancy.

The study was conducted in accordance with the principles outlined in the Declaration of Helsinki for biomedical research involving human subjects. The study protocol was approved by the institutional ethics committee. The requirement for informed consent was waived due to the retrospective nature of the study; therefore, informed consent was not obtained from the patients included in the study.

Following enrollment, patients underwent medical history review, physical examination, and assessment of demographic and pharmacological treatment parameters. The presence of hypertension, diabetes mellitus, smoking history, hyperlipidemia, and history of cerebrovascular disease was recorded. Baseline laboratory values prior to flecainide initiation were collected for all participants. These included complete blood count, serum blood urea nitrogen, creatinine, electrolytes (Na, K, Ca, and Mg), high-sensitivity C-reactive protein, total cholesterol, low-density lipoprotein cholesterol, high-density lipoprotein cholesterol, and triglycerides. All tests were performed using automated analyzers (Abbott Aeroset, Minneapolis, MN, USA) and standardized commercial kits (Abbott). Echocardiographic measurements were also obtained for all patients. Left atrial diameter and left ventricular ejection fraction (LVEF) were recorded using an EPIQ 7 ultrasound system (Philips Healthcare, Andover, MA, USA), with LVEF calculated automatically using the Simpson’s biplane method [25].

### 2.2. Twelve-Lead Electrocardiographic Evaluation

**Figure 1:**
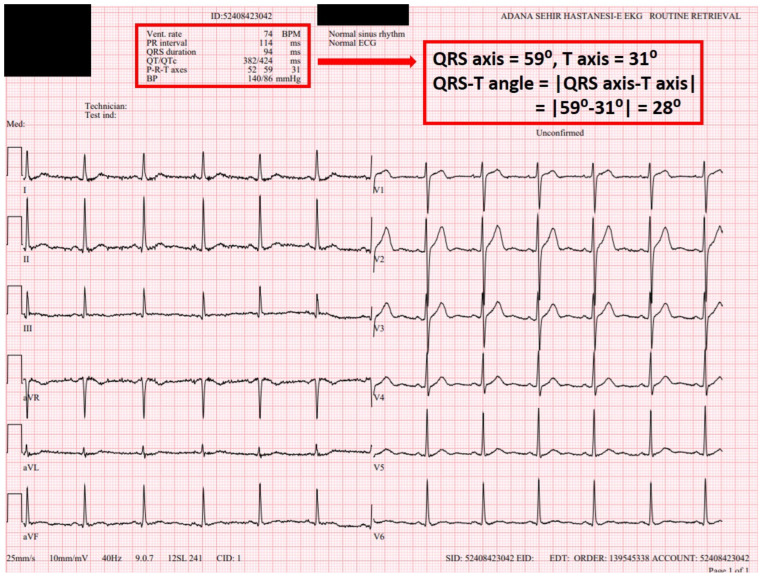
The 12-lead electrocardiogram (ECG) was obtained prior to the initiation of flecainide treatment in 55-year-old male patient with newly diagnosed paroxysmal atrial fibrillation (AF). HR (74 bpm), PR interval (114 ms), QRS duration (94 ± 9 ms), QT interval (382 ms), QTc interval (424 ms) and QRS–T angle (28) were measured.

**Figure 2:**
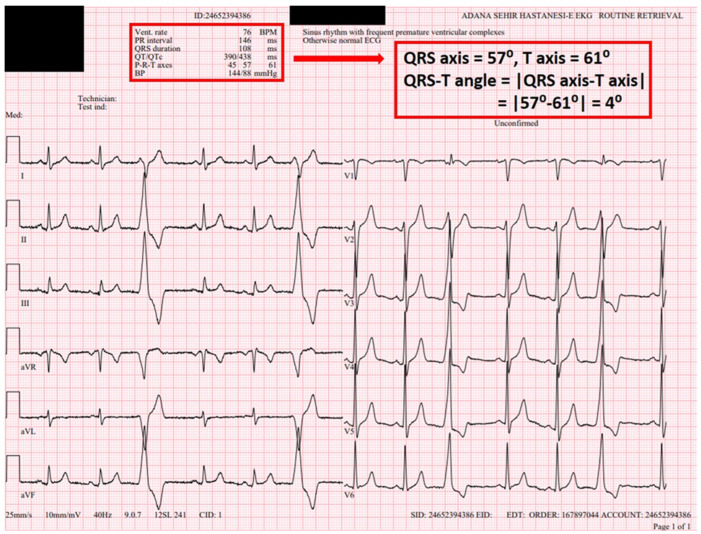
The 12-lead ECG was obtained prior to the initiation of flecainide treatment in 50-year-old male patient with premature ventricular contractions (PVCs). HR (76 bpm), PR interval (146 ms), QRS duration (108 ± 9 ms), QT interval (390 ms), QTc interval (438 ms) and QRS–T angle (4) were measured.

**Figure 3:**
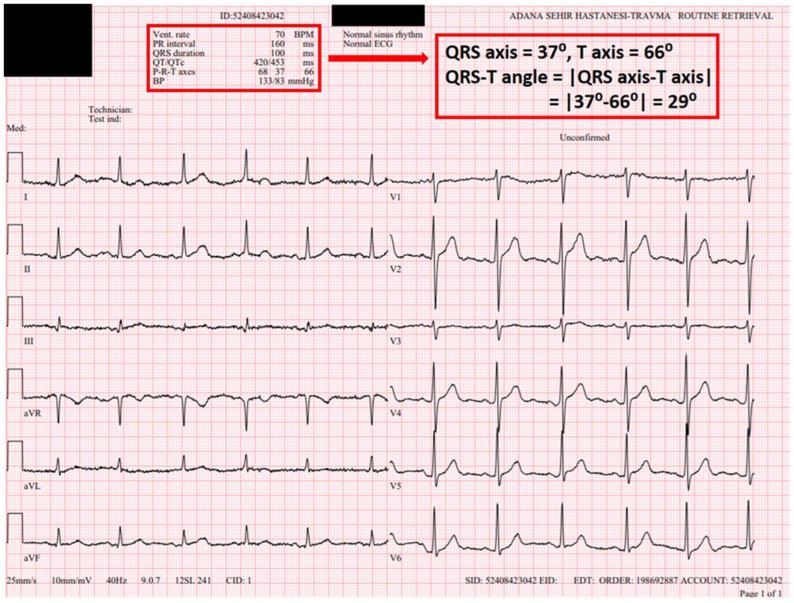
The 12-lead ECG was obtained 1 month after the initiation of flecainide treatment in the same AF patient (Figure 1). HR (70 bpm), PR interval (160 ms), QRS duration (100 ± 9 ms), QT interval (420 ms), QTc interval (453 ms) and QRS–T angle (29) were measured. Flecainide treatment was continued because the patient’s complaint improved and there were no significant ECG abnormalities.

**Figure 4:**
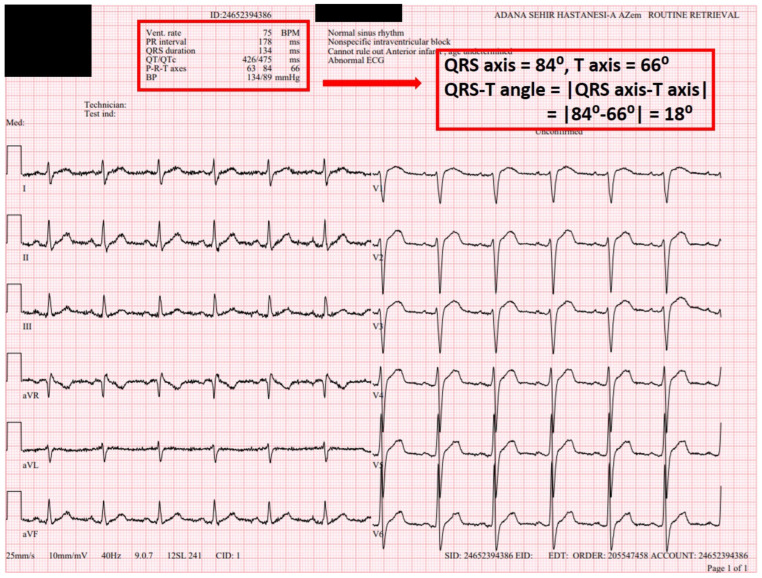
The 12-lead ECG was obtained 1 month after the initiation of flecainide treatment in the same PVC patient (Figure 2). HR (75 bpm), PR interval (178 ms), QRS duration (134 ms), QT interval (426 ms), QTc interval (475 ms) and QRS–T angle (18) were measured. Although the patient’s palpitations were relieved and PVCs disappeared, flecainide treatment was discontinued due to severe QRS and QTc prolongation.

All electrocardiographic recordings were obtained in sinus rhythm using a MAC 2000 ECG machine (GE Medical Systems Information Technologies, Inc., Milwaukee, WI, USA) with a standard paper speed of 25 mm/s and a calibration of 1 mV/10 mm. Before initiation of flecainide therapy, the following parameters were measured for each patient: heart rate (HR), PR interval, QRS duration, QT interval, corrected QT interval (QTc), JT interval, Tp–Te interval and frontal QRS–T angle (Figure 1 and Figure 2). The QT interval was measured from the onset of the QRS complex to the point where the T wave returned to the isoelectric line. The QTc was calculated using Bazett’s formula: QTc = QT/√(R–R interval). The Tp–Te interval was defined as the time from the peak of the T wave to the point where the T wave returned to the isoelectric baseline. Measurements were primarily performed from lead V5. If V5 was unsuitable for analysis (e.g., amplitude < 1.5 mm), leads V4 or V6 were used as alternatives. The QRS-T angle reflects the relationship between the ventricular depolarization vector and the repolarization vector and can be categorized into two types: (1) the spatial QRS-T angle [s(QRS-T)], defined as the angle between the QRS and T-wave vectors in three-dimensional (3D) space. The s(QRS-T) angle is measured now as the maximum magnitude of the spatial QRS and T vectors within a 3D QRS loop and T loop; however, its assessment requires specialized equipment. Accordingly, s(QRS-T) angle measurement was not performed in the present study. (2) The frontal QRS-T angle, which represents the projection of the spatial QRS-T angle onto the frontal plane, was calculated as the absolute difference between the QRS axis and the T axis. Unlike the spatial QRS-T angle, the frontal QRS-T angle is automatically computed by standard ECG devices—including the device used in our institution. For this reason, the frontal QRS-T angle was employed in the present study. At the 1-month follow-up visit, a standard 12-lead ECG was recorded again, and the same parameters (HR, PR and QRS durations, QT, QTc, JT, Tp–Te intervals and frontal QRS–T angle) were remeasured (Figure 3 and Figure 4). All ECGs recorded in sinus rhythm were independently reviewed in a blinded manner by two experienced electrophysiologists (MLA and FK), each with over 10 years of clinical electrophysiology experience and who evaluate ≥2000 arrhythmia patients annually. In the case of discrepancies between the two reviewers, a consensus was reached through consultation with a third senior electrophysiologist (MK).

### 2.3. Antiarrhythmic Treatment Initiation and Follow-Up

Prior to the initiation of flecainide therapy, all patients were evaluated for rhythm status. Those not in sinus rhythm underwent electrical cardioversion to restore sinus rhythm before starting treatment. For patients already in sinus rhythm, flecainide therapy was initiated directly. Before administration, each patient was carefully assessed for any contraindications or clinical conditions that would make flecainide use inappropriate. Flecainide was started at a standard dosage of 100 mg twice daily, in accordance with current clinical recommendations. In the present study, flecainide dose escalation was not performed during follow-up. All patients underwent 72 h ambulatory rhythm monitoring (Holter ECG) at least one month after inclusion in the study. Patients were followed at 1-month and 3-month intervals to evaluate for adverse drug effects and proarrhythmic events. Flecainide therapy was discontinued in the event of serious cardiac or non-cardiac side effects (Figure 4).

### 2.4. Statistical Analysis

All statistical analyses were performed using SPSS version 23.0 (SPSS for Windows 20.0, Chicago, IL, USA). Continuous variables were expressed as mean ± standard deviation (SD), while categorical variables were presented as frequencies and percentages. The distribution of continuous variables was assessed using the Kolmogorov–Smirnov test. To compare the changes in measurable ECG parameters before and after flecainide treatment, the paired *t*-test was used for normally distributed variables, and the Wilcoxon signed-rank test was used for non-normally distributed variables. A *p*-value of <0.05 was considered statistically significant for all comparisons.

## 3. Results

A total of 200 patients (97 females, 103 males; mean age: 55.6 ± 14.9 years) who were initiated on flecainide therapy for ventricular or supraventricular arrhythmias were included in the study. Fourteen patients with permanent atrial fibrillation were excluded from the study during the screening process. The frequency of atrial and ventricular arrhythmia included in the study was found to be 86% and 14%, respectively. For all participants, 12-lead ECGs were obtained immediately prior to flecainide initiation. The following parameters were measured: HR, PR interval, QRS duration, JT, QT, QTc and Tp–Te intervals and frontal QRS–T angle. These measurements were repeated at the 1-month follow-up visit after flecainide therapy. Cohen’s kappa values evaluating interobserver variability exceeded 90% for all ECG parameters (*p* < 0.001 for all comparisons), indicating excellent agreement.

Adverse effects—both cardiac and non-cardiac—were evaluated. Symptomatic ventricular or supraventricular arrhythmias were observed in seven patients, and four patients developed ECG abnormalities requiring discontinuation of treatment in the absence of arrhythmias. Non-cardiac side effects included: severe dizziness in two patients, visual dimming in one patient, severe headache in one patient, nausea in one patient, constipation in one patient, and fatigue in one patient. Due to these reasons, flecainide therapy was discontinued in 18 patients (9%) at the 1-month follow-up.

Demographic, clinical, and laboratory characteristics of the study population are presented in Table 1. The prevalence of cardiovascular risk factors was as follows: hypertension (41%), diabetes mellitus (16%), smoking (21%), hyperlipidemia (29%), and previous cerebrovascular events (4%).

Comparisons of ECG parameters before and after flecainide treatment are summarized in Table 2. HR significantly decreased from 78 ± 22 to 74 ± 15 bpm (*p* < 0.05). The PR interval increased from 148 ± 23 ms to 156 ± 29 ms, and the QRS duration increased from 89 ± 17 ms to 99 ± 19 ms (both *p* < 0.05). Additionally, significant increases were observed in the JT interval (326 ± 27 to 334 ± 26 ms), QT interval (416 ± 24 to 434 ± 24 ms), QTc interval (431 ± 24 to 447 ± 25 ms) and Tp–Te interval (84 ± 17 to 87 ± 18 ms) (*p* < 0.05 for all). No statistically significant change was detected in the frontal QRS–T angle. At the 1-month ECG follow-up, the QRS duration increased by more than 25% in eight patients, and QTc exceeded 480 ms in five patients (Figure 4).

## 4. Discussion

The primary finding of this study is that flecainide therapy in patients with atrial and ventricular arrhythmias did not result in a significant change in the frontal QRS–T angle. This finding is novel and represents, to our knowledge, the first study in the literature to specifically investigate this relationship. Another important finding of our study is the significant prolongation of the Tp–Te interval in patients treated with flecainide, which supports limited existing data on this topic. Furthermore, we demonstrated significant prolongation in key conduction and depolarization and repolarization parameters, including the QRS duration, JT interval, QT interval, and QTc interval—well-known side effects associated with flecainide therapy.

Over the past two decades, there has been little change in the range of antiarrhythmic drug options available. Flecainide is a class IC antiarrhythmic agent that exerts its effects by blocking the fast inward Na^+^ current during cardiac depolarization. Together with propafenone, it is considered a first-line option for the long-term treatment of ventricular and supraventricular arrhythmias in patients without structural heart disease [3,4]. Its limited use in clinical practice is partly attributed to the findings of the 1991 CAST trial, which demonstrated increased mortality among patients with ischemic heart disease receiving flecainide [26]. Therefore, thorough evaluation for contraindications is essential prior to initiating flecainide therapy. Additionally, cardiac and non-cardiac side effects occur in approximately 1–13% of patients [1].

Among the most critical ECG changes during flecainide therapy is QRS prolongation [1,24]. Some authors even consider QRS prolongation to be the primary marker of antiarrhythmic activity [1,23]. A 10–20% increase in QRS and QT durations is expected following initiation of the drug [1]. According to the European Society of Cardiology guidelines, if the QRS duration increases by ≥25% or if a left or right bundle branch block develops (QRS > 120 ms), discontinuation of therapy is recommended [3,4]. Others suggest reducing the dose by half in such cases [1]. In our study, we observed a significant increase in QRS duration consistent with the literature, and in eight patients, the QRS duration increased by more than 25% at the 1-month ECG follow-up, prompting discontinuation of treatment.

The effect of flecainide on the JT interval—a more isolated indicator of ventricular repolarization—remains controversial. While some studies have shown modest JT prolongation in approximately 4% of patients [20], others have reported no significant change [21,22]. At higher doses, flecainide may inhibit potassium channels in addition to sodium channels, contributing to prolonged repolarization [20]. Our findings are consistent with studies reporting JT prolongation as a reflection of extended action potential duration.

The QT interval is composed of the QRS duration plus the JT interval. Flecainide-induced prolongation of both components contributes to an overall 8–10% increase in QT and QTc durations [1,19,20,21,22]. In line with these findings, our study also demonstrated a significant prolongation in QT and QTc intervals, and five patients had QTc values exceeding 480 ms at 1-month follow-up, which led to treatment discontinuation.

The Tp–Te interval is associated with transmural dispersion of repolarization [27,28]. The Tp wave represents epicardial repolarization, while Te reflects the completion of myocardial repolarization [29]. Increased transmural dispersion of repolarization has been linked to a higher risk of ventricular arrhythmias [30]. Although few studies have investigated this, flecainide has been shown to prolong the Tp–Te interval, and our study confirms this finding.

Similarly, the QRS–T angle reflects transmural dispersion of repolarization, analogous to the Tp–Te interval. It quantifies the spatial relationship between the vectors of ventricular depolarization and repolarization and is defined as the absolute angular difference between the QRS axis and the T-wave axis [31]. An increased QRS–T angle is primarily associated with abnormal T-wave axis orientation and reflects maladaptive repolarization, which may predispose to malignant arrhythmias [32,33]. No prior studies have evaluated the effect of flecainide on the QRS–T angle. In our study, we observed that flecainide did not significantly alter this parameter. In the present study, the exact mechanism underlying the absence of a significant change in the QRS-T angle—despite flecainide therapy producing a substantial prolongation of QRS duration consistent with the existing literature—remains incompletely understood. It is well established that a close and robust relationship exists between the QRS duration and the QRS axis. We therefore hypothesize that the comparable effects of flecainide on both the QRS and T axes may have resulted in a proportional shift in each vector, thereby leaving the QRS-T angle unchanged. However, to our knowledge, no comparable studies or data addressing this specific question are currently available in the literature. Prospective studies enrolling larger patient populations are warranted to provide more definitive insight into this matter.

Flecainide is also associated with non-cardiac side effects, including hypotension, headache, dizziness, nausea, constipation, fatigue, and peripheral neuropathy [34]. Some of these effects may be mitigated when flecainide is co-administered with beta-blockers. In our study, seven patients discontinued treatment due to non-cardiac side effects: severe dizziness (n = 2), visual dimming (n = 1), severe headache (n = 1), nausea (n = 1), constipation (n = 1), and fatigue (n = 1). These findings underscore the importance of evaluating tolerability and considering beta-blocker co-therapy to minimize adverse effects.

## 5. Limitations

First and foremost, this study was designed as a retrospective, single-center investigation involving a relatively small number of patients. A prospective, multicenter study with a larger patient population would provide more robust and generalizable results. Before initiating flecainide therapy, it is essential to rule out contraindications through a comprehensive assessment including a 12-lead ECG, exercise stress testing, and echocardiography. In our study population, none of the patients had symptoms, signs, or a history suggestive of myocardial ischemia. However, exercise stress testing was not performed, which may represent a limitation in excluding latent ischemia. Flecainide-related cardiac and non-cardiac side effects are typically reported to begin within 14 days of treatment initiation and tend to become clinically evident by day 21 [19]. In our study, the initial follow-up and side effect evaluation were conducted at one month. It is possible that different results might have been observed had the assessment been performed on day 21 instead. In the present study, the effect of flecainide on the QRS-T angle was evaluated at the currently guideline-recommended maximum daily dose of 200 mg in all enrolled patients. The effect of the higher maximum daily dose of 400 mg on the QRS-T angle was not assessed.

## 6. Conclusions

In patients receiving flecainide therapy for atrial and ventricular arrhythmias, prolongation was observed in atrioventricular conduction, ventricular depolarization, and repolarization parameters as measured by ECG. However, no significant change was detected in the frontal QRS–T angle. This may be primarily attributed to the lack of effect of flecainide on transmural dispersion of repolarization. Nevertheless, further prospective, multicenter studies involving larger patient populations are warranted to confirm these findings.

## Figures and Tables

**Table 1:** Demographic, clinical, medical and laboratory data of the study population.

Variables	n = 200
Age (year, mean ± SD and minimum–maximum)	55.6 ± 14.5 and 20–86
Gender (Male), n	103 (52%)
Hypertension, n (%)	81 (41%)
Diabetes mellitus, n (%)	31 (16%)
Smoking, n (%)	42 (21%)
Hypercholesterolemia, n (%)	57 (29%)
Previous cerebrovascular accident, n (%)	7 (4%)
Flecainide for ventricular arrhythmia, n (%)	28 (14%)
Flecainide for supraventricular arrhythmia, n (%)	172 (86%)
Beta-blocker use, n (%)	100 (100%)
Statin use, n (%)	57 (29%)
ACEI or ARB use, n (%)	118 (59%)
White blood cell (10^3^/µL)	8.30 ± 2.5
Platelet count (10^3^/µL)	261 ± 83
Hemoglobin (g/dL)	13.4 ± 2.1
Creatinine (mg/dL)	0.82 ± 0.25
Blood urea nitrogen (mg/dL)	33.8 ± 16.1
Sodium (mEq/L)	139 ± 2.6
Potassium (mEq/L)	4.41 ± 0.39
Calcium (mg/dL)	9.31 ± 0.49
Magnesium (mg/dL)	1.98 ± 0.20
High-sensitivity C-reactive protein (mg/L)	4.66 ± 3.46
Left ventricular ejection fraction (%)	58.5 ± 4.55
Left atrial dimension (mm)	39.1 ± 4.36

The values were shown as mean ± standard deviation or n (%), ACEI: angiotensin-converting enzyme inhibitor, ARB: angiotensin receptor blocker.

**Table 2:** Comparison of 12-lead electrocardiographic measurements before and after flecainide therapy.

Variables	Measurements Before Flecainide n = 200	Measurements After Flecainide n = 200	t and Z Value	*p*
Heart rate (beat/min)	78 ± 22	74 ± 15	2.656 ^t^	**0.009** ^a^
PR duration (ms)	148 ± 23	156 ± 29	−4.544 ^t^	**<0.001** ^a^
QRS duration (ms)	89 ± 17	99 ± 19	−14.005 ^Z^	**<0.001** ^b^
QT (ms)	416 ± 24	434 ± 24	−11.233 ^t^	**<0.001** ^a^
QTc interval (ms)	431 ± 24	447 ± 25	−10.015 ^t^	**<0.001** ^a^
JT interval (ms)	326 ± 27	334 ± 26	−5.004 ^t^	**<0.001** ^a^
Tp–Te interval (ms)	84 ± 17	87 ± 18	−21.755 ^Z^	**<0.001** ^b^
QRS–T angle (°)	41 ± 38	40 ± 39	0.178 ^Z^	0.859 ^b^

a: Paired *t*-test; b: Wilcoxon test, Statistically significant *p* values are shown in bold.

## Data Availability

The data that support the findings of this study are available from the corresponding authors upon reasonable request.

## References

[1] Basza M., Maciejewski C., Bojanowicz W., Balsam P., Grabowski M., Mitkowski P., Kempa M., Kowalski O., Kalarus Z., Jaguszewski M. (2023). Flecainide in clinical practice. Cardiol. J..

[2] Arunachalam K., Alzahrani T. (2025). Flecainide. StatPearls [Internet].

[3] Zeppenfeld K., Tfelt-Hansen J., de Riva M., Winkel B.G., Behr E.R., Blom N.A., Charron P., Corrado D., Dagres N., de Chillou C. (2022). 2022 ESC Guidelines for the management of patients with ventricular arrhythmias the prevention of sudden cardiac death. Eur. Heart J..

[4] Van Gelder I.C., Rienstra M., Bunting K.V., Casado-Arroyo R., Caso V., Crijns H.J.G.M., De Potter T.J.R., Dwight J., Guasti L., Hanke T. (2024). 2024 ESC Guidelines for the management of atrial fibrillation developed in collaboration with the European Association for Cardio-Thoracic Surgery (EACTS). Eur. Heart J..

[5] Al-Khatib S.M., Stevenson W.G., Ackerman M.J., Bryant W.J., Callans D.J., Curtis A.B., Deal B.J., Dickfeld T., Field M.E., Fonarow G.C. (2018). 2017 AHA/ACC/HRS guideline for management of patients with ventricular arrhythmias and the prevention of sudden cardiac death: A Report of the American College of Cardiology/American Heart Association Task Force on Clinical Practice Guidelines and the Heart Rhythm Society. J. Am. Coll. Cardiol..

[6] Aro A.L., Huikuri H.V., Tikkanen J.T., Junttila M.J., Rissanen H.A., Reunanen A., Anttonen O. (2012). QRS-T angle as a predictor of sudden cardiac death in a middle-aged general population. Europace.

[7] Castro-Torres Y., Carmona-Puerta R., Katholi R.E. (2015). Ventricular repolarization markers for predicting malignant arrhythmias in clinical practice. World J. Clin. Cases.

[8] Garcia R., Schröder L.C., Tavernier M., Gand E., de Keizer J., Holkeri A., Eranti A., Bidegain N., Alos B., Junttila J. (2024). QRS-T angle: Is it a specific parameter associated with sudden cardiac death in type 2 diabetes? Results from the SURDIAGENE and the Mini-Finland prospective cohorts. Diabetologia.

[9] May O., Graversen C.B., Johansen M.Ø., Arildsen H. (2017). A large frontal QRS-T angle is a strong predictor of the long-term risk of myocardial infarction and all-cause mortality in the diabetic population. J. Diabetes Complicat..

[10] Apak O., Avci B.S., Apak O., Avci A., Tugcan M.O., Yildirim A., Koc M., Saler T. (2025). The relationship between primary hyperparathyroidism and frontal QRS-T angle. J. Electrocardiol..

[11] Elmas A.N., Fedai H., Toprak K., Tascanov M.B., Altıparmak I.H., Biçer A., Demirbag R., Tanrıverdi Z. (2024). The Association of Electrical Risk Score with Prognosis in Patients with Non-ST Elevation Myocardial Infarction Undergoing Coronary Angiography. Anatol. J. Cardiol..

[12] Han X., Chen Z., Wang Y., Zhang J., Zhang Y., Su Q., Pan Z., Sun J., Wang Y. (2021). Prognostic significance of QRS distortion and frontal QRS-T angle in patients with ST-elevation myocardial infarction. Int. J. Cardiol..

[13] Zorlu Ç., Açıkel B., Ömür S.E. (2025). Frontal Plane QRS—T Angle Is a Predictor of Ventricular Arrhythmia in Heart Failure with Preserved Ejection Fraction. Ann. Noninvasive Electrocardiol..

[14] Chen S., Hoss S., Zeniou V., Shauer A., Admon D., Zwas D.R., Lotan C., Keren A., Gotsman I. (2018). Electrocardiographic Predictors of Morbidity and Mortality in Patients with Acute Myocarditis: The Importance of QRS-T Angle. J. Card. Fail..

[15] Li S.N., Zhang X.L., Cai G.L., Lin R.W., Jiang H., Chen J.Z., Xu B., Huang W. (2016). Prognostic Significance of Frontal QRS-T Angle in Patients with Idiopathic Dilated Cardiomyopathy. Chin. Med. J..

[16] Balasubramaniyam N., Palaniswamy C., Aronow W.S., Khera S., Balasubramanian G., Harikrishnan P., Doshi J.V., Nabors C., Peterson S.J., Sule S. (2013). Association of corrected QT interval with long-term mortality in patients with syncope. Arch. Med. Sci..

[17] Österberg A.W., Jablonowski R., Östman-Smith I., Carlsson M., Schlegel T.T., Green H., Gunnarsson C., Fernlund E. (2025). Spatial QRS-T angle can indicate presence of myocardial fibrosis in pediatric and young adult patients with hypertrophic cardiomyopathy. J. Electrocardiol..

[18] Gotsman I., Keren A., Hellman Y., Banker J., Lotan C., Zwas D.R. (2013). Usefulness of electrocardiographic frontal QRS-T angle to predict increased morbidity and mortality in patients with chronic heart failure. Am. J. Cardiol..

[19] Paolini E., Stronati G., Guerra F., Capucci A. (2019). Flecainide: Electrophysiological properties, clinical indications, and practical aspects. Pharmacol. Res..

[20] Aliot E., Capucci A., Crijns H.J., Goette A., Tamargo J. (2011). Twenty-five years in the making: Flecainide is safe and effective for the management of atrial fibrillation. Europace.

[21] Sarubbi B., Ducceschi V., Briglia N., Mayer M.S., Santangelo L., Iacono A. (1998). Compared effects of sotalol, flecainide and propafenone on ventricular repolarization in patients free of underlying structural heart disease. Int. J. Cardiol..

[22] Sarubbi B., Ducceschi V., Briglia N., Esposito R., Mayer M.S., Scialdone A., Santangelo L., Iacono A. (1996). Sotalolo, propafenone e flecainide: Analisi comparata multiparametrica della ripolarizzazione ventricolare in soggetti esenti da cardiopatie organiche [Sotalol, propafenone, and flecainide: Compared multiparametric analysis of ventricular repolarization in subjects without organic cardiopathy]. Cardiologia.

[23] Andrikopoulos G.K., Pastromas S., Tzeis S. (2015). Flecainide: Current status and perspectives in arrhythmia management. World J. Cardiol..

[24] Morganroth J., Horowitz L.N. (1984). Flecainide: Its proarrhythmic effect and expected changes on the surface electrocardiogram. Am. J. Cardiol..

[25] Lang R.M., Bierig M., Devereux R.B., Flachskampf F.A., Foster E., Pellikka P.A., Picard M.H., Roman M.J., Seward J., Shanewise J.S. (2005). Recommendations for chamber quantification: A report from the American Society of Echocardiography’s Guidelines and Standards Committee and the Chamber Quantification Writing Group, developed in conjunction with the European Association of Echocardiography, a branch of the European Society of Cardiology. J. Am. Soc. Echocardiogr..

[26] Akiyama T., Pawitan Y., Greenberg H., Kuo C.S., Reynolds-Haertle R.A. (1991). Increased risk of death and cardiac arrest from encainide and flecainide in patients after non-Q-wave acute myocardial infarction in the Cardiac Arrhythmia Suppression Trial. CAST Investigators. Am. J. Cardiol..

[27] Medina-Ravell V.A., Lankipalli R.S., Yan G.X., Antzelevitch C., Medina-Malpica N.A., Medina-Malpica O.A., Droogan C., Kowey P.R. (2003). Effect of epicardial or biventricular pacing to prolong QT interval and increase transmural dispersion of repolarization: Does resynchronization therapy pose a risk for patients predisposed to long QT or torsade de pointes?. Circulation.

[28] Lubinski A., Lewicka-Nowak E., Kempa M., Baczynska A.M., Romanowska I., Swiatecka G. (1998). New insight into repolarization abnormalities in patients with congenital long QT syndrome: The increased transmural dispersion of repolarization. Pacing Clin. Electrophysiol..

[29] Antzelevitch C., Shimizu W., Yan G.X., Sicouri S., Weissenburger J., Nesterenko V.V., Burashnikov A., Di Diego J., Saffitz J., Thomas G.P. (1999). The M cell: Its contribution to the ECG and to normal and abnormal electrical function of the heart. J. Cardiovasc. Electrophysiol..

[30] Barbhaiya C., Po J.R., Hanon S., Schweitzer P. (2013). Tpeak—Tend and Tpeak—Tend/QT ratio as markers of ventricular arrhythmia risk in cardiac resynchronization therapy patients. Pacing Clin. Electrophysiol..

[31] Pavri B.B., Hillis M.B., Subacius H., Brumberg G.E., Schaechter A., Levine J.H., Kadish A., Defibrillators in Nonischemic Cardiomyopathy Treatment Evaluation (DEFINITE) Investigators (2008). Prognostic value and temporal behavior of the planar QRS-T angle in patients with nonischemic cardiomyopathy. Circulation.

[32] Chaudhry U., Cortez D., Platonov P.G., Carlson J., Borgquist R. (2020). Vectorcardiography Findings Are Associated with Recurrent Ventricular Arrhythmias and Mortality in Patients with Heart Failure Treated with Implantable Cardioverter-Defibrillator Device. Cardiology.

[33] Rozen G., Kobo R., Beinart R., Feldman S., Sapunar M., Luria D., Eldar M., Levitan J., Glikson M. (2013). Multipole analysis of heart rate variability as a predictor of imminent ventricular arrhythmias in ICD patients. Pacing Clin. Electrophysiol..

[34] Wehling M. (2002). Meta-analysis of flecainide safety in patients with supraventricular arrhythmias. Arzneimittelforschung.

